# RNA-Seq Reveals Gastrointestinal Transcriptome Dynamics in Preweaning Yak Calves Fed Concentrate Supplements

**DOI:** 10.3390/ani16091329

**Published:** 2026-04-27

**Authors:** Ya-Nan Zhou, Xin-Ya Bie, Jin-Quan Yuan, Yin Wang, Wen-Jie Guo, Shu-Jie Liu, Zhan-Hong Cui

**Affiliations:** Qinghai Academy of Animal Science and Veterinary Medicine, Qinghai University, Xining 810016, China; yanan_zhou0312@163.com (Y.-N.Z.); biexinya0304@126.com (X.-Y.B.); yjq13147637931@163.com (J.-Q.Y.); jiajia0306518@126.com (Y.W.); nyz0801@126.com (W.-J.G.); mkylshj@126.com (S.-J.L.)

**Keywords:** concentrate supplementation, yak calves, gastrointestinal, gastrointestinal immunity, transcriptome

## Abstract

The objective of this study was to investigate the effects of supplemental starter feeding on the development of the ruminal epithelium in suckling yak calves using transcriptomic analysis. Twenty healthy one-month-old male yak calves with similar body weights were selected and randomly assigned to two groups. The pre-feeding adaptation period lasted 14 days, followed by a 120-day experimental feeding period. At the end of the trial, five calves from each group were slaughtered, and samples of abomasum tissue and ruminal contents were collected for subsequent analyses. The results demonstrated that early concentrate supplementation markedly increased the final body weight and ruminal NH_3_-N concentration of calves in the RAS group compared with the control (RA) group (*p* < 0.05). Similarly, dry matter intake and ruminal microbial protein (MCP) content were significantly higher in the RAS group (*p* < 0.05). In contrast, the concentration of acetic acid in ruminal fluid was significantly higher in the RA group, whereas valeric acid concentration was higher in the RAS group. Furthermore, ruminal TNF-α, TNF-γ, and IL-2 concentrations were significantly elevated in the RAS group (*p* < 0.05), suggesting enhanced ruminal immune function. Transcriptomic analysis revealed that both up- and down-regulated gene expression contributed to the morphological development and overall health of the ruminal epithelium. Up-regulated genes were enriched in pathways related to chemical carcinogenesis, cytochrome P450 metabolism, steroid hormone biosynthesis, retinol metabolism, ascorbate and aldarate metabolism, drug metabolism-cytochrome P450, pentose and glucuronate interconversions, ovarian steroidogenesis, and porphyrin and chlorophyll metabolism. Conversely, down-regulated genes were mainly associated with cytokine–cytokine receptor interactions, mineral absorption, arachidonic acid metabolism, and viral protein interactions with cytokine receptors. Overall, early supplementation with concentrate feed enhanced the expression of genes associated with ruminal epithelial development, improved immune responses, and promoted better growth performance in suckling yak calves.

## Simple Summary

Recent research has demonstrated that early nutritional intervention in yak calves can influence developmental processes through gastrointestinal transcriptomic reprogramming. Our study revealed that concentrate supplementation significantly reshapes the functional development of the rumen–intestinal axis, characterized by the upregulation of key metabolic pathways, such as cytochrome P450 metabolism and steroid hormone biosynthesis, and the downregulation of inflammatory pathways, including cytokine-receptor interaction. This nutritional programming effect not only enhanced dry matter intake and rumen fermentation efficiency but also strengthened mucosal immune barrier function by modulating the expression of immune factors such as TNF-α and IL-2. Notably, calves receiving early nutritional intervention exhibited marked activation of genes related to rumen morphological development (e.g., *CYR61*, *KRT18*), while the upregulated expression of the lipid metabolism gene THRS suggested a long-term regulatory potential for energy partitioning. These findings provide a molecular-level theoretical basis for optimizing yak calf rearing through early nutritional strategies on the Qinghai-Tibet Plateau, indicating that precise nutritional interventions during lactation may program gastrointestinal development in ruminants through epigenetic mechanisms.

## 1. Introduction

Yak (*Bos grunniens*), a unique livestock species native to the Qinghai–Tibet Plateau, is often referred to as the “boat of the plateau” and an “omnipotent livestock” due to its remarkable adaptability to harsh high-altitude environments. The yak industry has expanded rapidly in recent years, with the total yak population in Qinghai Province reaching 6.52 million head and an annual meat output of 62,600 tons by the end of 2021 [1]. Previous studies have demonstrated the importance of early nutritional management in ruminants. Connor et al. [2] reported that calves fed milk replacer combined with concentrate exhibited superior ruminal papilla development compared with those receiving milk replacer alone or milk replacer plus forage. For yak calves, alfalfa hay and concentrate serve as essential carbohydrate sources that play key roles in promoting growth performance and gastrointestinal development [3]. Studies by Baldwin and Xie [4,5] further confirmed that early concentrate supplementation markedly accelerates ruminal development in young ruminants. The ruminal epithelium is fundamental not only for nutrient absorption but also for protecting animals from mechanical injury and pathogen invasion [6]. The ruminal barrier consists of three major components: the microbial, physical, and immune barriers. The microbial barrier, composed of diverse commensal microorganisms, inhibits pathogen colonization by competing for nutrients, secreting antibacterial metabolites, and modifying environmental conditions [7]. The physical barrier, formed by tight junctions between epithelial cells, prevents toxins and pathogens from translocating into the bloodstream, whereas the immune barrier comprises mucosa-associated immune cells and cytokines that maintain ruminal homeostasis [8]. The integrity of this epithelial barrier is thus critical for maintaining ruminal health and function, protecting epithelial cells from injury, and limiting microbial invasion [9]. On the Qinghai–Tibet Plateau, the ruminal epithelial development of yak calves fed with concentrate is essential for achieving high-quality breeding outcomes and elucidating the mechanisms underlying ruminal growth. Substantial evidence suggests that increasing dietary concentrate levels promotes bone and rumen development while enhancing the expression of genes regulating ruminal epithelial growth [10,11,12]. Moreover, in yak calves, the development of the ruminal stratum corneum stimulated by forage and solid feed accelerates growth performance and gastrointestinal function, facilitating the rapid establishment of digestive capacity [13,14].

In ruminants, ingested feed is first fermented in the rumen to produce volatile fatty acids and other metabolites through microbial fermentation. Unfermented substrates, along with ruminal microbes, pass into the intestine, where further digestion and absorption occur, supplying nutrients and energy to the animal [15]. The intestinal tract, therefore, plays a vital role in digestion, absorption, and immune defense. The intestinal mucosal immune system serves as the first line of defense against pathogens [16]. For yak calves, supplementation with solid feed during lactation stimulates gastrointestinal development, whereas traditional milk-only feeding fails to meet nutritional demands and limits digestive tract maturation [17,18].

Given the crucial role of intestinal development in growth performance, the present study aimed to investigate the effects of concentrate supplementation on the morphological and functional development of the gastrointestinal tract in suckling yak calves.

## 2. Materials and Methods

### 2.1. Animal Ethics

All animal procedures were conducted in accordance with the Guidelines for the Care and Use of Laboratory Animals issued by the State Administration of Experimental Animals (Ministry of Science and Technology, Beijing, China, revised in 2004).

### 2.2. Animals and Experimental Design

**Figure 1:**
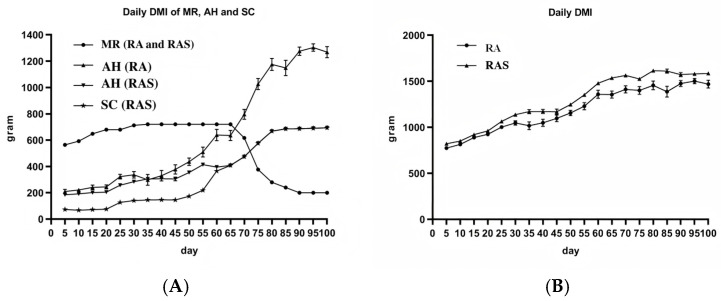
Daily DMI of each diet (**A**) and daily total DMI (**B**) of yak calves in group RA and RAS. Note: MR, milk replacer; AH, alfalfa hay; SC, concentrate.

This experiment was conducted in Haiyan County, Haibei Tibetan Autonomous Prefecture, Qinghai Province, China, using a single-factor completely randomized design. The pre-feeding adaptation period lasted 14 days, followed by a 120-day formal trial. Twenty healthy male yak calves aged 30 days [(30 ± 3) d] with similar body weights were selected and randomly assigned to two groups (*n* = 10 per group): a control group (RA) and a supplemented group (RAS). The experiment consisted of a 30-day pre-feeding period followed by a 100-day formal experimental period. During the pre-feeding period, all calves were gradually adapted to milk replacer and alfalfa hay, while calves in the RAS group were additionally adapted to concentrate. The formal trial commenced thereafter. During the experimental period, calves in the RA group were fed milk replacer and alfalfa hay, whereas calves in the RAS group were fed milk replacer, alfalfa hay, and concentrate. The daily amount of milk replacer was identical for both groups and was provided three times daily (morning, noon, and evening). The milk replacer was thoroughly mixed with warm water (boiled and cooled to approximately 42 °C) at a ratio of 1:5 (*w*/*v*), poured into feeding bottles, and placed on bottle racks to allow the calves to suckle voluntarily. The dry matter (DM) offered from alfalfa hay in the RA group and from alfalfa hay plus concentrate in the RAS group was kept equal and provided twice daily (morning and evening). From days 1 to 50, alfalfa hay and concentrate in the RAS group were offered at a DM ratio of 2:1. From days 51 to 100, this ratio was adjusted to 1:1 based on actual conditions. During the feeding trial, the amount of each feed offered was recorded daily, and feed refusals for each calf were measured before the morning feeding on the following day. Daily dry matter intake (DMI) of each feed (Figure 1A) and total daily DMI (Figure 1B) were calculated for both groups. Subsequently, the average daily DMI of each feed, average daily nutrient intake (on a DM basis), and total DMI were calculated for each group over the entire experimental period. All calves were housed individually in pens with access to both indoor and outdoor areas, ensuring sufficient space and sunlight exposure. Water was provided ad libitum. The pens were cleaned and disinfected weekly. Immediately after slaughter, the rumen, jejunum, and colon were dissected, weighed, and sampled.

Feed samples were collected weekly during the experimental period. For each feed type, subsamples were obtained from multiple locations within the feed batch and across different feeding days to ensure representativeness. All subsamples were pooled and homogenized to obtain a composite sample for each feed type. The samples were stored at −20 °C until further analysis. Before chemical analysis, samples were dried at 65 °C for 48 h, ground to pass through a 1 mm sieve, and thoroughly mixed. Nutrient composition was determined according to AOAC (Association of Official Analytical Chemists) procedures. DM, CP, EE, and ash were analyzed using standard AOAC methods, while NDF and ADF were determined following the Van Soest method with the use of heat-stable amylase and expressed exclusive of residual ash. Starter feed refers to the concentrate mixture used in this study, which was formulated to meet the nutritional requirements of preweaning calves and consisted primarily of corn, soybean meal, bran, and other ingredients. The nutrient compositions of milk replacer, alfalfa hay, and concentrate are shown in Table 1 and Table 2.

### 2.3. Sample Collection

The slaughter was conducted following humane protocols to ensure animal welfare and sample integrity. Animals were first rendered unconscious (via captive bolt stunning), followed by exsanguination via the carotid arteries and jugular veins. This procedure, lasting 3 to 5 min, is efficient and minimizes pre-slaughter stress, thereby preventing physiological artifacts in the collected tissues while maintaining the structural integrity of the organs. Following slaughter, the abdominal cavity was opened to remove the viscera, and the rumen was promptly isolated. Ruminal fluid was filtered through four layers of gauze, transferred into centrifuge tubes, and immediately frozen in liquid nitrogen. Tissue samples (3 cm × 2 cm) were collected from the ruminal ventral sac, jejunum, and colon, rinsed with sterile saline, and dissected into mucosal and muscular layers using sterilized instruments. The ruminal epithelial, jejunal, and colonic mucosal tissues were placed in 5 mL cryovials and stored at −80 °C until analysis.

### 2.4. Determination of Indicators and Methods

#### 2.4.1. Growth Performance and Ruminal Fermentation

On the first and last days of the feeding trial, each yak calf was weighed in the morning before feeding. Body measurements, including body height, body length (oblique length), chest girth, and cannon bone circumference, were recorded using a measuring tape and measuring stick. The average daily gain (ADG) and feed-to-gain ratio (F/G) were calculated for both groups. In addition, the weights of the rumen, jejunum, and colon were recorded after slaughter. The main parameters for ruminal fermentation, including NH_3_-N concentration [19], microbial protein concentration (MCP) [20], and volatile fatty acid (VFA) concentration [21], were determined using specific methods. The NH_3_-N concentration was measured using an Ultraviolet spectrophotometer (TU-1810, Beijing Purkinje General Instrument Co., Ltd., Beijing, China) with a colorimetric method. The MCP concentration was determined through the Coomassie brilliant blue method. Lastly, the VFA concentration was determined using gas chromatography (GC-2014, Shimadzu Corporation, Kyoto, Japan).

#### 2.4.2. Ruminal Immune Factors

Approximately 0.5 g of mucosal tissue was homogenized in phosphate-buffered saline (PBS; pH 7.4, 1:9 *w*/*v*) under liquid nitrogen and centrifuged at 4 °C for 10 min. The supernatant was collected and stored at −20 °C until analysis. The concentrations of IL-1β, IL-2, IL-4, IL-6, IL-10, TNF-α and TNF-γ in the ruminal were determined using commercial ELISA kits (Jiangsu Jingmei Biotechnology Co., Ltd., Yancheng, Jiangsu, China) following the manufacturer’s instructions.

#### 2.4.3. Rumen and Intestinal Transcriptomic Analysis

Transcriptomic analysis was performed to examine gene expression profiles in the ruminal epithelium, jejunal, and colonic mucosa of yak calves. The jejunum and colon samples from the RA group were designated as AJ and AC, respectively, while those from the RAS group were labeled ASJ and ASC. Approximately 100 mg of each frozen tissue was homogenized in liquid nitrogen, and total RNA was extracted using TRIzol reagent (Invitrogen, Carlsbad, CA, USA) with DNase I treatment to remove genomic DNA contamination. RNA concentration and purity were assessed using a NanoDrop 1000 spectrophotometer (Thermo Fisher Scientific, Waltham, MA, USA), and RNA integrity was verified using an Agilent 2100 Bioanalyzer(Agilent Technologies, Santa Clara, CA, USA). Only high-quality RNA samples (RIN ≥ 7.0) were used for library construction. cDNA libraries were prepared using the Illumina TruSeq RNA kit (Illumina, San Diego, CA, USA) following standard protocols and sequenced on an Illumina NovaSeq platform (150 bp paired-end) by Nuohe Zhiyuan Biotechnology Co., Ltd. (Beijing, China). Raw reads were filtered to remove adapters and low-quality sequences using fastp (v0.23.2), and clean reads were aligned to the yak reference genome (*Bos mutus*, BosGru_v2.0) using HISAT2 (v2.2.1). Gene-level counts were generated using featureCounts (v2.0.3) . Gene expression levels were quantified as FPKM, and genes with FPKM > 1 in at least one sample were considered expressed. Differential expression analysis was conducted using DESeq2 (v1.38.3) , with thresholds of |log2 fold change| ≥ 1 and FDR < 0.05 to define differentially expressed genes (DEGs). Principal component analysis (PCA) was performed to assess global transcriptional variation among samples. DEGs were visualized by hierarchical clustering and heatmaps using Euclidean distance. Functional enrichment of DEGs was conducted using clusterProfiler (v4.6.2) based on Gene Ontology (GO) and Kyoto Encyclopedia of Genes and Genomes (KEGG) databases. Pathways with *p* < 0.05 were considered significantly enriched, highlighting key biological processes and signaling pathways involved in ruminal and intestinal development in yak calves.

Quantitative real-time PCR (qRT-PCR) was used to validate selected RNA-Seq results on a QuantStudio 6 Flex Real-Time PCR System (Applied Biosystems, Foster City, CA, USA). Primers were synthesized by Bao Biological Engineering Co., Ltd. (Shanghai, China) and are listed in Table 3.

Each 20 μL reaction mixture contained 10 μL SYBR Green PCR Master Mix, 200 nM of each primer, and 1–10 ng of cDNA template. The thermal cycling conditions consisted of an initial denaturation at 95 °C for 30 s, followed by 40 cycles of 95 °C for 5 s and 60 °C for 31 s. Relative gene expression levels were normalized to GAPDH as an internal reference gene and calculated using the 2^−ΔΔCt^ method.

### 2.5. Data Processing and Analysis

Data on growth performance, ruminal fermentation, and immune indices were processed using Microsoft Excel 2016 and analyzed by one-way ANOVA in SPSS 20.0 (IBM Corp., Armonk, NY, USA). Pearson correlation analysis was applied to assess relationships between transcriptomic and phenotypic parameters. Statistical significance was set at *p* < 0.05.

## 3. Results

### 3.1. Growth Performance and Ruminal Fermentation

Growth performance and ruminal fermentation parameters were determined based on the research group’s previous findings [22]. Following early concentrate supplementation, calves in the RAS group exhibited a significant increase in final body weight and ruminal NH_3_-N concentration (*p* < 0.01). Moreover, dry matter intake, average daily gain (ADG), heart girth, cannon circumference and ruminal microbial protein (MCP) content were significantly higher in the RAS group compared with the RA group (*p* < 0.05). In contrast, the concentration of acetic acid in ruminal fluid was significantly greater in the RA group, whereas valeric acid concentration was significantly higher in the RAS group (*p* < 0.05) (Table 4).

### 3.2. Ruminal Immune Factor

As presented in Table 5, the ruminal concentrations of TNF-α and TNF-γ in the RAS group were significantly higher than those in the RA group (*p* < 0.05). Similarly, the ruminal IL-2 content was markedly elevated in the RAS group compared with the RA group (*p* < 0.05).

### 3.3. Ruminal Transcriptional Sequencing Analysis

#### 3.3.1. Analysis of Differential Gene Expression in the Rumen

**Figure 2:**
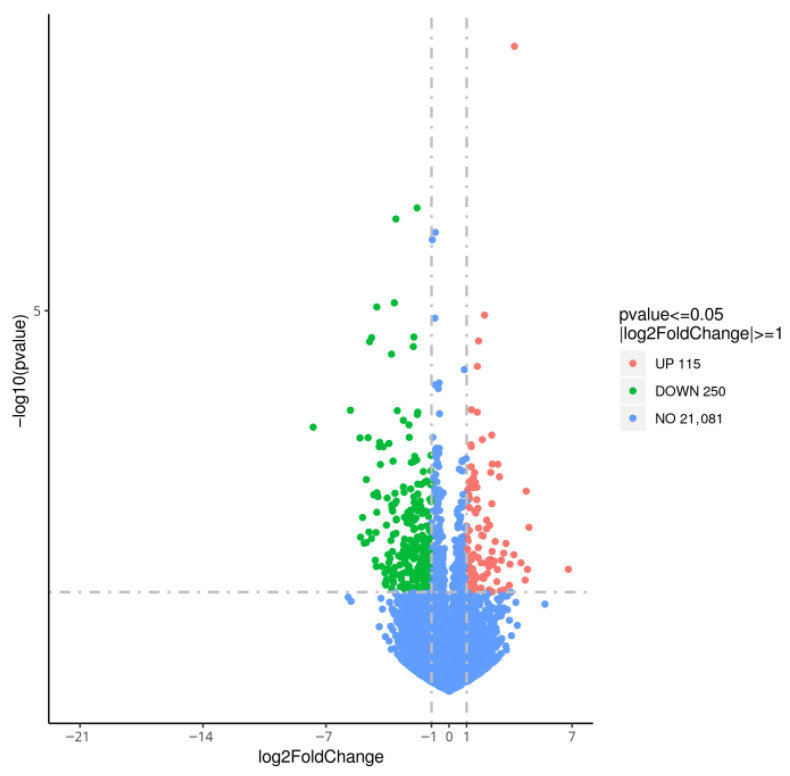
Volcanic map of differential expression genes of ruminal tissue.

Based on the screening criteria of FDR < 0.05 and |log_2_(fold change)| ≥ 1, a total of 365 differentially expressed genes (DEGs) were identified in the rumen of yak calves in the RAS group compared with the RA group. Among these, 115 genes were significantly upregulated and 250 were downregulated (Figure 2). The top 20 upregulated genes are listed in Table 6.

#### 3.3.2. GO Functional Enrichment Analysis

**Figure 3:**
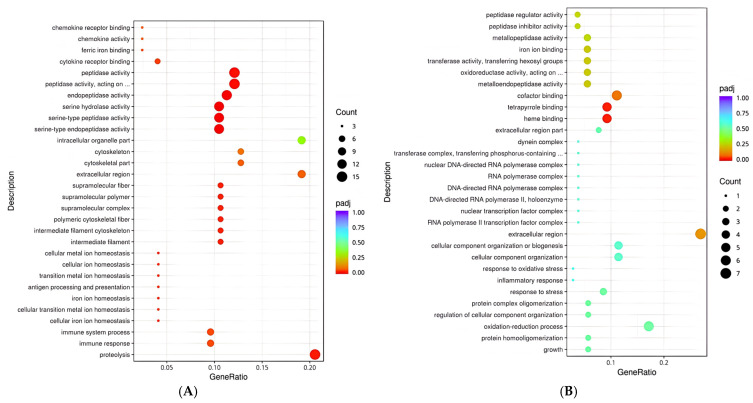
Differential gene ontology enrichment bubble map for up (**A**) and down (**B**) regulated gen. Note: In (**A**), “peptidase activity, acting on …” corresponds to “peptidase activity, acting on L-amino acid peptides”, and in (**B**), “oxidoreductase activity, acting on …” corresponds to “oxidoreductase activity, acting on paired donors, with incorporation or reduction of molecular oxygen”.

Gene Ontology (GO) functional enrichment analysis (Figure 3) revealed that the upregulated genes in the RAS group were significantly enriched in terms related to heme binding and tetrapyrrole binding (*p* < 0.05). Other enriched functions included cofactor binding, extracellular region, iron ion binding, transferase activity, oxidoreductase activity, metallopeptidase activity, peptidase inhibitor activity, peptidase regulator activity, oxidation–reduction processes, cellular component organization, and protein homogeneity (*p* > 0.05). In contrast, downregulated genes were significantly enriched in functions associated with peptidase activity, endopeptidase activity, serine hydrolase activity, and serine-type endopeptidase activity (*p* < 0.05). Moreover, these downregulated genes were closely linked to biological processes such as proteolysis, immune response, and immune system processes (*p* < 0.05). At the cellular component level, significant enrichment was observed in intermediate filaments, intermediate filament cytoskeleton, and polymeric cytoskeletal fibers (*p* < 0.05). Overall, these findings suggest that the differential expression of genes associated with structural, enzymatic, and immune functions contributes to the morphological development and improved rumen health of yak calves.

#### 3.3.3. KEGG Pathway Enrichment Analysis

**Figure 4:**
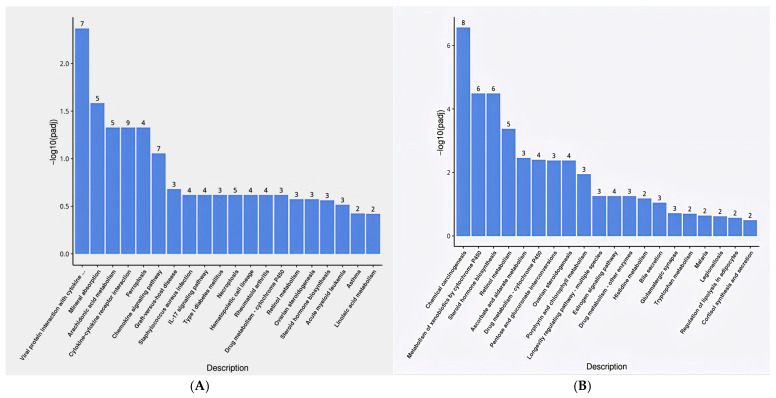
KEGG pathways significantly enriched for up (**A**) and down (**B**) regulated genes. Note: In (**A**), “Viral protein interaction with cytokine …” corresponds to “Viral protein interaction with cytokine and cytokine receptor.”

The differential gene sets were subjected to KEGG pathway enrichment analysis (Figure 4). The results indicated that the up-regulated gene enrichment pathways included Chemical carcinogenesis, metabolism of xenobiotics by cytochrome P450, Steroid hormone biosynthesis, Retinol metabolism, Ascorbate, alternate metabolism, drug metabolism-cytochrome P450, Pentose and gluconate interconversion, Ovarian steroidogenesis, and blocking the pathway of porphyrin and chlorophyll (*p* < 0.05). Conversely, the down-regulated gene enrichment pathways encompassed cytokine-cytokine receptor interaction, Mineral absorption, Arachidonic acid metabolism, Viral protein interaction with cytokine and cytokine receptor, and iron apoptosis pathway (*p* < 0.05).

#### 3.3.4. Quantitative PCR Verification

**Figure 5:**
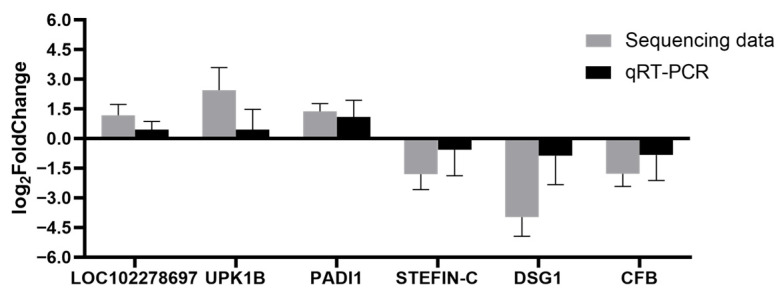
Real-time quantitative PCR to verify the accuracy of sequencing results.

The sequencing results can accurately and reliably reflect gene expression, as demonstrated by the consistent verification results of three randomly selected up-regulated genes (*UPK1B*, *PADI1*, *LOC102278697*) and three randomly selected down-regulated genes (*STEFIN-C*, *DSG1*, *CFB*), as shown in Figure 5.

### 3.4. Intestinal Transcriptional Sequencing Analysis

#### 3.4.1. Analysis of Differential Gene Expression in the Intestine

**Figure 6:**
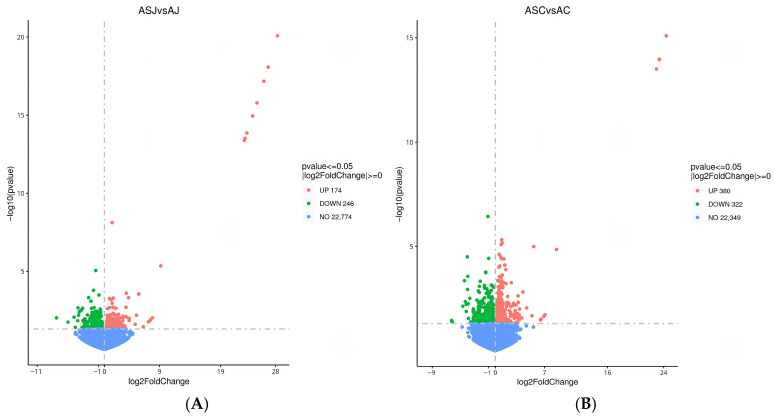
Volcano map of differentially expressed genes jejunum (**A**) and colon (**B**).

A total of 420 differentially expressed genes were screened for the jejunum, with 174 genes up-regulated and 246 genes down-regulated in the RAS group compared to the RA group (Figure 6A). Similarly, for the colon, a total of 702 differentially expressed genes were screened, with 380 genes up-regulated and 322 genes down-regulated in the RAS group relative to the RA group (Figure 6B). In Table 7 and Table 8, the top 20 differentially expressed genes in the jejunum and colon are presented.

#### 3.4.2. GO Functional Enrichment Analysis

**Figure 7:**
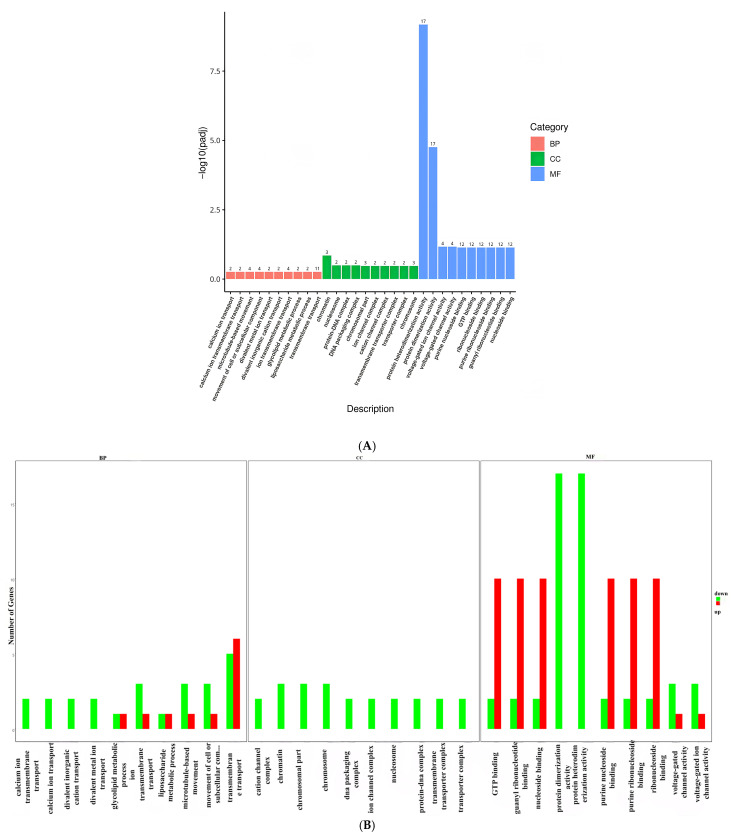

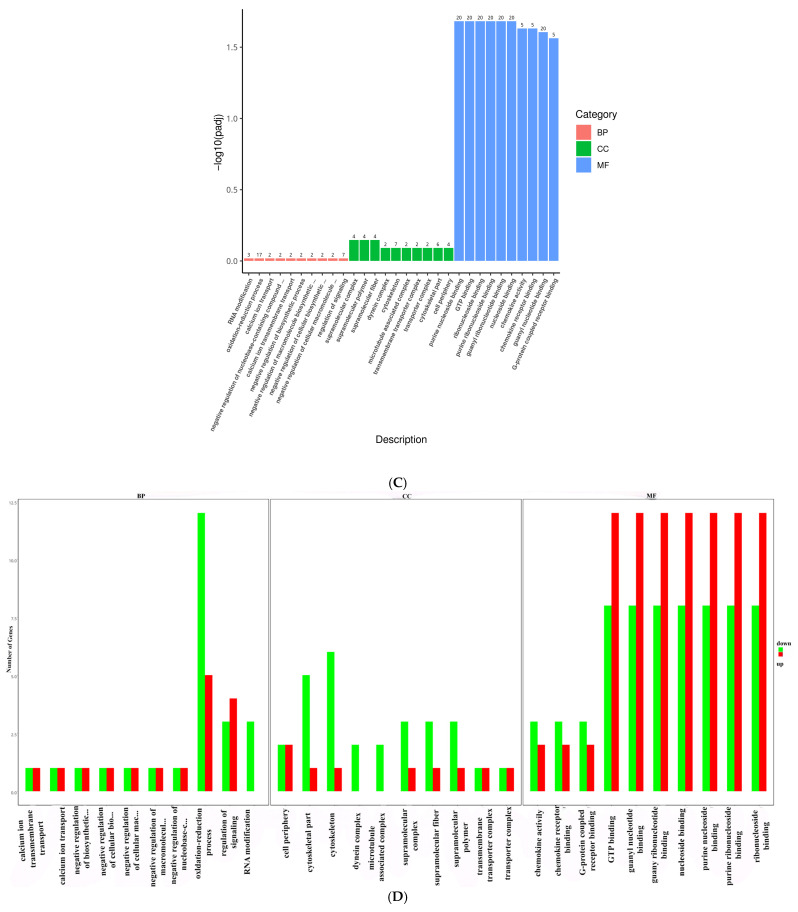
Analysis of jejunal and colonic GO functional enrichment (**A**,**C**) enrichment significance (**B**,**D**) Number of enriched genes. Note: In (**B**), “movement of cell or subcellular com …” corresponds to “movement of cell or subcellular component”. In (**C**), “negative regulation of nucleobase-containing compound …” corresponds to “negative regulation of nucleobase-containing compound metabolic process”, “negative regulation of macromolecule biosynthetic process” corresponds to “negative regulation of nucleobase-containing compound metabolic process”, “negative regulation of cellular biosynthetic …” corresponds to “negative regulation of cellular biosynthetic process”. In (**D**), “negative regulation of biosynthetic …” corresponds to “negative regulation of biosynthetic process”, “negative regulation of cellularbio …” corresponds to “negative regulation of cellular biosynthetic process”, “negative regulation of cellularmac …” corresponds to “negative regulation of cellular macromolecule metabolic process”, “negative regulation of macromolecul …” corresponds to “negative regulation of macromolecule metabolic process”, “negative regulation of nucleobase-c …” corresponds to “negative regulation of nucleobase-containing compound metabolic process”.

For the jejunum, differentially expressed genes were found to be enriched in Biological Process (BP), Cellular Component (CC), and Molecular Function (MF) categories based on GO function enrichment analysis. Specifically, there were 195 enriched functions in the Biological Process category, 60 in the Cellular Component category, and 167 in the Molecular Function category. A bar graph of the top 30 significantly enriched functions (Figure 7A) showed that differentially expressed genes were mainly enriched in the Molecular Function category, particularly in functions such as GTP binding, guanyl ribonucleotide binding, nucleoside binding, protein heterodimerization activity, protein dimerization activity, and various types of nucleoside and ribonucleoside binding. The most abundant differentially expressed gene in the Biological Process category was related to transmembrane transport (Figure 7B). Moreover, functions in the Cellular Component category solely consisted of down-regulated differentially expressed genes. In addition, both protein heterodimerization activity and protein dimerization activity functions exclusively contained down-regulated differentially expressed genes.

For the colon, GO function enrichment analysis revealed that differentially expressed genes were enriched in 245 functions in the Biological Process category, 53 functions in the Cellular Component category, and 194 functions in the Molecular Function category. The significantly enriched functions in the Molecular Function class one included purine nucleoside binding, GTP binding, ribonucleoside binding, and 10 others (Figure 7C). Similar to the results in the jejunum, differentially expressed genes in the colon were predominantly enriched in the Molecular Function category, specifically in functions such as GTP binding, guanyl nucleotide binding, and guanine nucleotide binding. Additionally, within the Biological Process category, differentially expressed genes were notably abundant in oxidation–reduction processes and regulation signaling (Figure 7D).

#### 3.4.3. KEGG Pathway Enrichment Analysis

**Figure 8:**
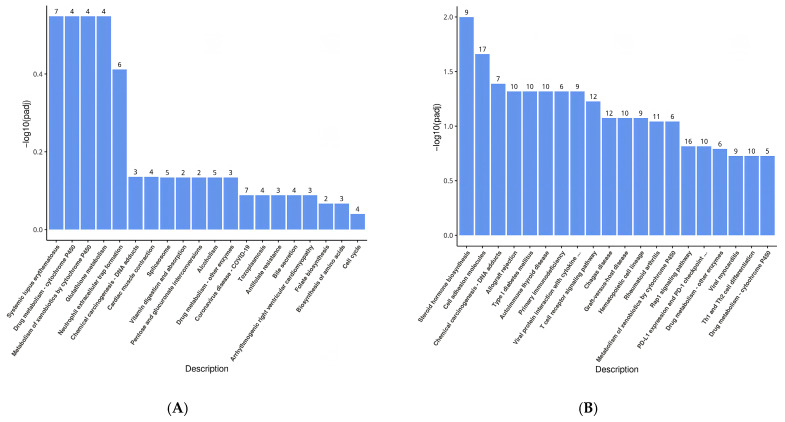
KEGG pathway enrichment analysis jejunum (**A**) and colon (**B**).

Differentially expressed genes underwent KEGG pathway enrichment analysis, as shown in Figure 8. The bar chart displays the top 20 enriched pathways, selected based on the level of significance. In the jejunum, the enriched pathways encompassed Glutathione metabolism, Vitamin digestion and absorption, Antifolate resistance, bile secretion, folic acid biosynthesis, and biosynthesis of amino acids. Similarly, the enrichment pathways in the colon consisted of Steroid hormone biosynthesis, Cell adhesion molecules, T cell receptor signaling pathway, and Th1 and Th2 cell differentiation, among others.

#### 3.4.4. Quantitative PCR Verification

**Figure 9:**
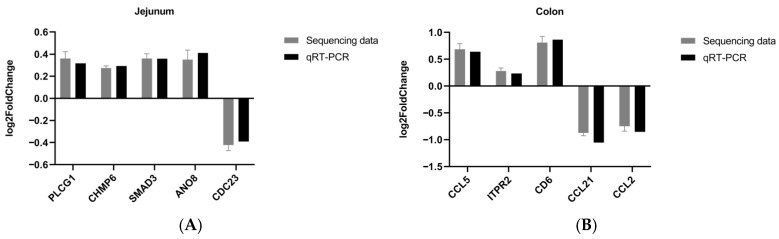
Real-time quantitative PCR to verify the accuracy of sequencing results ((**A**) jejunum, (**B**) colon).

**Figure 10:**
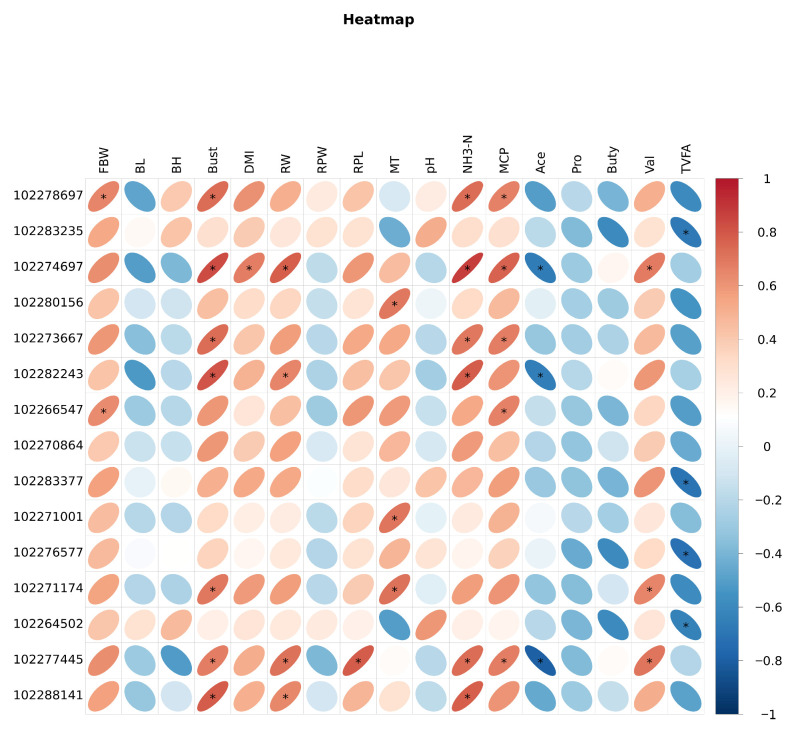
Correlation Analysis of Growth Performance, Rumen Fermentation Parameters, and Rumen Transcriptome. RBW: Final body weight; DMI: Dry matter intake; RW: Ruminal weight; RPW: Ruminal papilla width; RPL: Ruminal papilla length; MT: Muscle thickness; NH_3_N: NH_3_-N; MCP: Microbiota crude protein; Ace: Acetate; Pro: Propionate; But: Butyrate; Ibu: Isobutyrate; Val: Valerate; Iva: Isovalerate. “*” indicates *p* < 0.05.

The accuracy of the sequencing data was further verified by real-time quantitative PCR (Figure 9). Five differentially expressed genes were randomly selected. The sequencing results showed the same trend as the quantitative results as shown in Figure 10, indicating a high accuracy of the sequencing data.

### 3.5. Correlation Analysis of Growth Performance, Ruminal Fermentation Parameters, and Ruminal Transcriptome

Figure 10 presents the correlation analysis of growth performance, ruminal fermentation parameters, and ruminal transcriptomics of yak calves. The body weight of the calves showed a significant positive correlation with *LOC102278697* and *ADAMTS1* (*p* < 0.05). Moreover, dry matter intake exhibited a positive correlation with *PADI1* (*p* < 0.05), while NH_3_-N was positively correlated with MCP and the majority of up-regulated genes (*p* < 0.05). Conversely, acetic acid and total volatile fatty acids demonstrated a negative correlation with the up-regulated genes (*p* > 0.05).

## 4. Discussion

With advances in animal nutrition, scientific supplementation of concentrate not only enhances the reproductive performance of female animals but also mitigates grassland degradation caused by overgrazing, thereby promoting the sustainable development of animal husbandry. In plateau regions, nutrient deficiencies and harsh environmental conditions severely constrain the survival rate of yak calves. Therefore, this study evaluated the effects of early concentrate supplementation on yak calves. The results of the present study showed that early supplementation significantly improved growth performance, with average weight gain reaching 45.7 kg. This finding was consistent with the report of Huang [23], who observed similar improvements in yak calves during the cold season. Previous studies demonstrated that feeding pelleted concentrates before and after weaning enhances dry matter digestibility and growth performance in calves [24], and that supplementary feeding markedly increases dry matter intake. Similarly, the present study observed significant increases in body height and chest girth in the supplemented group, consistent with earlier findings [24,25]. Cui [26] also reported that milk replacer combined with concentrate supplementation significantly improves dry matter intake and weight gain in yak calves, supporting the results of this study. Under identical management and environmental conditions, calves not receiving concentrate exhibited lower body weights, likely due to their reliance on milk replacer and alfalfa hay as the primary feed sources. Alfalfa hay provides limited nutrients and contains high fiber levels, which may reduce digestibility and nutrient utilization, thereby constraining growth and development. Collectively, these findings indicate that early concentrate supplementation effectively enhances growth performance and economic efficiency in grazing yaks. However, further research is warranted to elucidate the underlying mechanisms regulating the growth response to scientific concentrate supplementation.

The cultivation mode of milk replacer and concentrate, under the influence of the early cultivation mode, can relieve weaning stress and improve rumen development level. It has been found that both alfalfa and concentrate can change the health and immunity of ruminants’ digestive tract [27,28]. The rumen development of calves is slow at birth, and it mainly relies on the digestion and absorption processes of the abomasum and intestines. As calves age and begin to consume solid feed, the production of volatile fatty acids (VFA) through microbial fermentation in the rumen stimulates the rapid development of the rumen. This leads to the gradual establishment of immune metabolism function in the digestive system. The fermentation of ruminal ruminants, which occurs in the largest fermentation place, produces VFA that directly stimulates the development of the ruminal epithelium [29]. The level of NH_3_-N and microbial protein (MCP) in ruminant ruminal microbes indicates their fermentation state. NH3-N maintains equilibrium in the rumen and enhances the synthesis of MCP by rumen microbes, serving as an indicator of ruminal microbial activity [30]. To ensure that ruminal microorganisms make full use of nitrogen sources, the content of non-fiber carbohydrates such as monosaccharides and disaccharides in the diet should be appropriately increased. The utilization of NH_3_-N by ruminal microorganisms is largely influenced by dietary nutritional levels, particularly the balance between nitrogen and fermentable carbohydrates [31]. In the present study, calves in the RAS group exhibited a significantly higher ruminal NH_3_-N concentration compared to the RA group, which appears inconsistent with previous findings that increasing the concentrate-to-forage ratio typically reduces ruminal NH_3_-N concentration [32]. This discrepancy may be explained by several factors. First, the rumen of preweaning calves is not fully developed, and the capacity of ruminal microorganisms to assimilate ammonia nitrogen into microbial protein is still limited. Therefore, although concentrate supplementation increases the availability of fermentable substrates, the efficiency of NH_3_-N utilization may remain low, resulting in its accumulation in the rumen. Second, the inclusion of concentrate likely increased the intake of rumen degradable protein, leading to enhanced protein degradation and ammonia production. When the rate of ammonia production exceeds microbial utilization capacity, NH_3_-N accumulates in the rumen [33]. Additionally, differences in feed intake between groups may have contributed to increased nitrogen input in the RAS group, further elevating NH_3_-N levels. Despite the higher NH_3_-N concentration, the significantly increased MCP content in the RAS group suggests that concentrate supplementation improved the overall microbial growth environment. This indicates that early supplementation with concentrate may enhance ruminal microbial activity and partially promote nitrogen utilization, even though complete synchronization between energy and nitrogen supply has not yet been achieved in preweaning calves. Short-chain fatty acids produced by ruminal microorganisms degrading cellulose provide 70% to 80% of the energy required by ruminants [34]. Acetic acid, among these fatty acids, is absorbed by the ruminal epithelium for involvement in the tricarboxylic acid cycle and fat synthesis. It can also be converted into butyric acid through a reaction to participate in ketone body formation, thereby promoting fat accumulation in animals [35]. According to Liu [36], an increase in the proportion of roughage in the diet leads to an increase in the concentration of acetic acid in ruminal fermentation, with cellulolytic bacteria being the predominant microbial species. The levels of neutral-washing fiber and acid-washing fiber were higher in the calves of the RA group who only consumed alfalfa hay in this experiment. This resulted in a significant increase in ruminal acetic acid concentration. This finding aligns with Wang’s study [37] where they increased the level of acid-washing fiber in the diet and observed an increase in acetic acid concentration. The higher acetate concentration in group RA indicates a more fibrolytic fermentation pattern dominated by fiber-degrading microorganisms, which primarily produce acetate as the main volatile fatty acid. In contrast, the increase in valerate concentration in group RAS suggests enhanced metabolic activity associated with mixed carbohydrate and amino acid fermentation under concentrate supplementation. However, no significant difference was observed in the acetate-to-propionate ratio between the two groups, indicating that the dietary supplementation level of concentrate was insufficient to substantially shift the overall rumen fermentation pattern from acetate-dominated to propionate-dominated fermentation during the experimental period. This may be attributed to the buffering capacity and functional stability of rumen microbial communities, which maintain fermentation homeostasis despite moderate dietary changes.

For young ruminants, the development of the rumen epithelium and the fermentation activity of rumen microorganisms directly influence their growth performance. Improvements in growth performance are often accompanied by enhanced rumen development. Lymphocytes distributed throughout the gastrointestinal tract play a vital role in resisting pathogens by producing various cytokines and exerting cytotoxic effects. Therefore, healthy gastrointestinal development is crucial for realizing the productive potential of ruminants in adulthood [38]. The results revealed that concentrated feed in yak calves enhanced the content of IL-2, TNF-α, and IFN-γ, facilitating the elimination of bacteria and the clearance of virus-infected cells, as compared to the control group. According to Wasilewska [39], the expression of TNF-α and IFN-γ genes in the gastrointestinal tract of lambs is closely related to the rumen. This suggests that microbial changes caused by different diets may have an impact on the secretion of cytokines in the two groups. In another study conducted by Cheng [40], it was found that feeding Bacillus subtilis to Hu sheep can lead to an increase in the contents of globulin, globulin, IFN-γ, IL-2, and IL-6, thereby enhancing immunity. Lin [41] reported that the expression of rumen-related genes can be induced by butyric acid in the rumen, thereby promoting the differentiation and proliferation of ruminal epithelial cells.

Through their research on rumen development in young animals using transcriptomics, Sun [42] discovered that the metabolism of VFA directly contributes to energy production, which drives the development of the rumen wall. This finding provides evidence that supplementing hay promotes immune function establishment, whereas concentrate aids in the transportation and metabolism of nutrients. These processes are essential for the biological development of the rumen. To investigate the impact of gastrointestinal development on immune function, we measured the transcription of rumen. With the gradual intake of solid concentrate, rumen development is gradually improved [13]. Studies have shown that there is no significant difference in gene expression in ruminal tissue between calves of 2 and 6 weeks old when they are breastfed with their mothers or only fed with milk replacer [2,5]. Increasing the level of energy and protein in the diet has been shown to increase the expression of *NHE1*, *NHE2*, and *NHE3* genes in the ruminal epithelium of goats, as demonstrated in a study examining the effect of a high-concentration diet on rumen development. These genes, namely the Na+/H+ exchanger genes *NHE1* and *NHE3*, play a role in regulating the concentration and pH of ruminal volatile fatty acids. Supplementing alfalfa before weaning has also been found to induce changes in the expression of certain ruminal epithelial genes, specifically those involved in glucose homeostasis, triglyceride biosynthesis, and the epithelial growth factor receptor signaling pathway. This supplementation has been shown to promote early ruminal wall development and alleviate weaning stress [43]. Penner [44] and Tayyab [45] have suggested a different perspective from previous feeding experiments on cows and sheep, claiming that the expression of *NHE* in the ruminal epithelium of ruminants remains unaffected by dietary composition and nutritional levels. However, Etschmann [46] have provided evidence demonstrating that the diet does indeed regulate the transport of sodium ions through *NHE*.

In this study, we found that there were 365 differentially expressed genes in the ruminal tissues of yak calves. Significantly up-regulated genes related to rumen development included *CYR61* and *KRT18*, while the *THRSP* genes associated with growth were also up-regulated. *CYR61*, a coding protein, is classified as an Insulin-like growth factor binding protein (IGF), and it promotes cysteine production in blood vessels. Increasing the levels of non-fibrous carbohydrate (NFC) and neutral detergent fiber in the diet can increase the gene expression of Insulin growth factor I (IGF-I), IGF-I primarily acts on the cell surface to promote protein and nucleic acid production in the carbohydrate metabolism pathway [47]. Several studies have demonstrated that IGF regulates the growth and development of ruminal epithelium [47,48,49]. Zhang [48] identified several related genes, including *KRT36*, *TGMK*, *OVOL1*, *DSP*, and *SPINK5*, that promote ruminal epithelial development. KRT18, an intermediate filament protein, is a gene protein that induces cell proliferation. The expression of the thyroid hormone-induced liver protein (*THRSP*) and the *UCP3* genes was found to be up-regulated in high-marbled beef, according to the results. This suggests that the level of adipogenic factors in beef is higher in different grades of marbled beef [47,48,50]. *THRSP* is known to be directly proportional to fat deposition in muscle. The significant up-regulation of the *THRSP* gene indicates that concentrate has a positive regulatory effect on the growth performance of calves. Although this study did not explore the meat quality of calves fed with concentrate, it is suggested that concentrate could promote the molecular functions of heme binding and tetrapyrrole binding through the genes *LOC102282243*, *LOC102283377*, *LOC102283235*, *PTGS2*, and *LOC102279228*. These genes may serve as potential markers affecting rumen development. Additionally, studies comparing natural grazing with supplementary feeding of yak calves have observed changes in the genes affecting rumen development in the supplementary feeding group. GO enrichment analysis further reveals that supplementary feeding has led to changes in metabolic pathways related to ruminal epithelial development. Through transcriptomic sequencing analysis, Loughlin [51] made an interesting discovery regarding the expressions of calves in various signaling pathways, such as the G protein-coupled receptor signaling pathway, cytokine signaling pathway, ion transmembrane transport, and others. Importantly, these findings shed light on potential approaches for reducing the weaning stress experienced by calves. In Baldwin et al.’s study [4], additives in the bull diet were observed to impact cholesterol metabolism, amino acid metabolism signal pathways, fat metabolism, carbohydrate metabolism, and small molecule biosynthesis pathways. The technical means of transcriptomics not only helps explore the differential gene expression among different treatment groups but also provides the theoretical basis for exploring the metabolic pathways involved by functional genes. A total of 39 differential genes play a role in these metabolic pathways. KEGG analysis showed that the pathways of significant enrichment of differential genes mainly include chemical carcinogenesis, the interaction between cytokines and receptors, metabolism of retinol, nutrient absorption of minerals, and steroid hormone biosynthesis. It was found that supplementing alfalfa hay inhibited the signal pathway of tumor necrosis factor, and the expression of inflammatory factors related to rumen development also decreased [52,53]. According to KEGG analysis, the alteration of differential genes due to early concentrate supplementation may result from the interaction between metabolism and cytokines, subsequently influencing the quality of ruminal epithelium development.

Analysis of the colon groups showed that immune-related genes like *ITGAD*, *GPR55*, *FAIM2*, *CD247*, *P2RY11*, and *CD6* were differentially expressed. In particular, ITGAD was found to encode integrin α-D, which plays a role in enhancing immune function through the phagocytosis of blood-borne pathogens and senescent erythrocytes [54]. A novel finding for the treatment of metabolic disorders is the regulation of nutrient metabolism by *GPR55*, which encodes a G protein-coupled receptor. Rudolf Schicho et al. [55] discovered that the *GPR55* gene is expressed in the gastrointestinal tract, and its agonists have been shown to reduce intestinal inflammation. Another study by Henstridge et al. [56]. further supports this, indicating that *GPR55* can be a potential target for the treatment of metabolic disorders. The *FAIM2* gene encodes an anti-apoptotic protein, the expression of which is influenced by diet [57]. In addition to this, the GPR55 gene is expressed not only on endothelial cells of the gastrointestinal tract but also on interosseous neurons, which may play a role in intestinal motility. *CD247* is associated with immunity. Reduced expression of the *CD247* gene leads to abnormal T lymphocyte activation and low immune function [58]. Ollila et al. [59] found that *p2RY11* has associations with immune function and may enhance intercellular adhesion. *CD6* is an antigen found on differentiated T cells [60], indicating that supplementing with concentrate is likely to enhance the immune response of the *CD6* subpopulation of T cells. Han Xiaoying [61] conducted a study on goats, in which they were fed with varying levels of ruminal degradable starch. The results showed that high levels of degradable starch had a significant impact on the expression of cecum tight junction protein genes, consequently affecting the epithelial barrier. Furthermore, the study identified *ACADSB*, *NCOA2*, and *GALNT16* as genes associated with nutrient digestion and metabolism. NCOA2 regulates lipid metabolism [62] and can positively regulate the secretion of bile acids into the intestine. One study found that bile acid transporter protein gene expression was significantly reduced in mice lacking this gene [63]. *ACADSB* is associated with short-chain/branch-chain fatty acid metabolism [64] and, more specifically, is the first step in the mitochondrial β-oxidation reaction [65]. The genes related to intestinal immunity and nutrient digestion and absorption were significantly upregulated in the supplemented concentrate group. Additionally, *GALNT16*, which is involved in lipid metabolism [66]. and *SLC16A6*, a gene that regulates monocarboxylic acid transport in tissues, were also upregulated [67]. However, the specific impact of *GALNT16* on lipid metabolism remains unclear. Furthermore, *IGFBP2* was significantly upregulated in the supplemented concentrate group. *IGF2*, an important gene associated with intestinal tissue development, has physiological functions such as promoting growth and stimulating adipocyte proliferation. It plays a crucial role in animal body weight and tissue development. On the other hand, *IGFBP2*, a significant binding protein for IGFs, regulates the activity of IGF2 and TGF-β. Thus, it indirectly affects animal body weight and tissue development [68]. Supplementation with concentrate affects intestinal development and animal growth by influencing immunity, nutrient digestion and absorption, and expression of developmental genes. The differential gene expression in the jejunum and colon groups, categorically enriched in biological processes, mainly relates to nutrient transport, metabolism, redox processes, and regulatory signaling. These processes, in turn, regulate intestinal function and affect intestinal development. The KEGG enrichment pathway of differentially expressed genes in the jejunum and colon primarily relates to nutrient digestion and absorption as well as immunity. Supplementation with concentrate has been found to mainly affect the digestion and absorption of nutrients, as well as immunity, thus facilitating the development of intestinal tissues and functions. Cui Zhanhong [26] discovered that the differentially expressed gene KEGG enrichment pathways, such as the T cell receptor signaling pathway, natural killer cell-mediated cytotoxicity pathway, and Th1 and Th2 cell differentiation, are influenced by concentrate supplementation, indicating its impact on various immune cell functions.

The yak calves supplemented with concentrate showed increased intake of protein and fat, resulting in rapid growth, as revealed through correlation analysis between phenotypic data and transcriptomics. Additionally, KEGG pathway enrichment analysis indicated that the genes in metabolic pathways were significantly enriched and positively correlated with growth performance. This enrichment was found to benefit the digestion and absorption of nutrients, as well as the synthesis pathways of pentose and glucosaccharase. Moreover, it was observed that the supplementation of concentrate enhanced the digestion and absorption of protein and fat in yak calves. Interestingly, in the rumen, volatile fatty acids were found to be low in yak calves supplemented with concentrate, suggesting that microorganisms primarily played a role in digestion and decomposition.

## 5. Conclusions

The results showed that early feeding concentrate could improve the immune ability of yak calves. This finding was further supported by the results of transcriptomics analysis. Additionally, the experiment concluded that supplemental concentrate feeding during lactation promoted gastrointestinal tract development, improved gastrointestinal immune function, and enhanced the digestion and absorption of nutrients in yak calves.

## Figures and Tables

**Table 1:** Nutrient levels of milk replacer and alfalfa hay (DM basis).

Items	Milk Replacer	Alfalfa Hay
Dry matter DM, % as fresh	95.00	93.80
Crude protein, %	26.24	12.50
Ether extract, %	27.79	0.90
Neutral detergent fiber, %	—	56.45
Acid detergent fiber, %	—	40.40
Ca, %	2.50	0.98
P, %	1.40	0.18
Ca/P	1.96	5.70

Note: Nutrient levels were measured values, the same as Table 2.

**Table 2:** Composition and nutrient levels of starter feed.

Items	Starter Feed
Ingredients (% DM basis)	
Corn, %	46.50
Soybean meal, %	27.00
Bran, %	9.50
Expanded soybean meal, %	8.00
Dried whey, %	5.00
Limestone, %	1.60
Premixture (1), %	1.00
Fat power, %	0.50
CaHPO_4_, %	0.50
NaCl, %	0.40
Nutrient levels	
DM, % as fresh	87.90
Crude protein, %	20.00
Ether extract, %	4.70
Neutral detergent fiber, %	10.90
Acid detergent fiber, %	4.10
Ca, %	0.80
P, %	0.45
Ca/P	1.78

Note: (1) The premix provided the following per kg of diets: Fe 22.1 mg, Mn 9.82 mg, Cu 2.25 mg, Zn 27.0 mg, Se 0.19 mg, I 0.54 mg, Co 0.09 mg, VE 4 000 IU, VD 30 000 IU, VA 30 000 IU.

**Table 3:** Sequences of forward and reverse primers of differential genes used for real-time PCR of rumen, jejunum and colon.

GenBank Accession No. (*Bos grunniens*)	Target Genes	Primer Sequence (5′-3′)	Production Size (bp)
XM_005904123.3	*GAPDH*	F: CATCACTGCCACCCAGAAGAC	335
		R: AGTGAGTGTCGCTGTTGAAGTCG	
XM_005918765.1	*LOC102278697*	F: GCAGGTGTGTAACCCCATCA	182
		R: AGCCTTGGATCCCAACAGTC	
XM_005915432.2	*STEFIN-C*	F: GTTCTCAGATACTCAGGATGCCACA	172
		R: ATCCTCGTCCACTTGAACCTTG	
XM_005910876.2	*PADI1*	F: GTCTCAGGAACGACAACCTCTATG	102
		R: GGATGTCCACGATGTCATGCTC	
XM_005913245.2	*UPK1B*	F: CCCATCAGACTGGCAGAAATACA	255
		R: AGGAGAACCCAGAAAGTCCAACA	
XM_005909876.3	*DSG1*	F: TAACGCAAATACACTGGTGATGG	112
		R: CGGTGAATCTGAGGGTTCTTGTC	
XM_005917654.2	*CFB*	F: GTCCTTCTGGCTTCTACCCATACC	108
		R: ATTCAGCCCTCTTGACAATCTTTCT	
XM_005906543.3	*PLCG1*	F: AAAAGGTGCTTGGGGACACA	146
		R: CAGTGCTGCACTTTGCCATT	
XM_005912345.2	*CHMP6*	F: CGTAAGAAACAGAGCCGGGT	138
		R: GAAGGGGCTCTGAAGGAACC	
XM_005908765.3	*SMAD3*	F: AACACTAACTTCCCTGCCGG	176
		R: CGGCAAACTCCTGGTTGTTG	
XM_005916234.2	*ANO8*	F: ACGCTGTTCTTGGAGGAGTG	162
		R: TGGCCAGCATCTCTTTCAGG	
XM_005911234.2	*CDC23*	F: TAAGCGGGACTACAGAGCCT	144
		R: GAGAAACTCTGCGTGTGGGA	
XM_005907654.2	*CCL5*	F: TGCTTCTGCCTCCCCATATG	127
		R: AGCGTTGATGTACTCTCGCA	
XM_005914567.3	*ITPR2*	F: ATGGGGGTGAAGATGTGCTG	191
		R: GAGGCTTTCTGGGCTGGATT	
XM_005918234.2	*CD6*	F: GCGGTTCAACAACTCCAACC	172
		R: GCACCTCTTCCAGTCCTTCC	
XM_005909432.2	*CCL21*	F: ATGGCCATGGCTCAGTCAC	163
		R: TATGGCCCTTTTGAGATCTCAGT	
XM_005906789.2	*CCL2*	F: CTCCTGTGCCTGCTACTCAC	154
		R: CTGCTTGGGGTCTGCACATA	

Note: The accession numbers correspond to representative RefSeq predicted transcripts (XM_) retrieved from the *Bos grunniens* reference genome (NCBI, BosGru v2.0).

**Table 4:** Effects of supplementary starter feeding on dry matter intake and weight gain of yak calves in the suckling stage.

Items	Groups		*p*-Value
	RA	RAS	
DMI of MR (g/d)	551.40 ± 0.00	551.40 ± 0.00	1.000
DMI of AH (g/d)	639.68 ± 26.38 ^a^	414.05 ± 4.56 ^b^	<0.001
DMI of SC (g/d)	-	331.35 ± 4.56	-
Total DMI (g/d)	1191.08 ± 26.38 ^b^	1296.80 ± 9.13 ^a^	0.001
Final Body Weight, kg	72.07 ± 2.47 ^B^	80.58 ± 1.26 ^A^	<0.001
Total weight gain, kg	39.62 ± 1.67	45.70 ± 3.89	0.077
ADG (g)	426.68 ± 8.89	449.23 ± 10.65	0.121
F/G	2.86 ± 0.07	2.96 ± 0.06	0.304
Body height (cm)	78.80 ± 1.00	79.40 ± 1.06	0.685
Body length (cm)	90.70 ± 1.42	88.70 ± 1.11	0.282
Heart girth (cm)	104.70 ± 0.87 ^b^	114.40 ± 0.99 ^a^	<0.001
Cannon circumference (cm)	13.40 ± 0.16 ^b^	14.10 ± 0.18 ^a^	0.010
NH_3_-N, mg/dL	2.46 ± 0.28 ^B^	3.29 ± 0.23 ^A^	0.001
MCP, mg/dL	13.10 ± 0.96 ^b^	17.20 ± 1.13 ^a^	0.013
Acetate, mmol/L	35.78 ± 3.51 ^a^	29.60 ± 1.05 ^b^	0.034
Propionate, mmol/L	9.39 ± 1.29	8.28 ± 1.07	0.232
Butyrate, mmol/L	5.41 ± 1.55	4.62 ± 1.18	0.389
Valerate, mmol/L	3.58 ± 0.43 ^b^	4.23 ± 0.05 ^a^	0.021
Acetate to propionate ratio	3.74 ± 0.10	3.45 ± 0.15	0.383
Total volatile fatty acids, mmol/L	57.40 ± 5.27	50.23 ± 5.25	0.081

Note: “-” indicates that the corresponding feed was not provided in this treatment. In the same row, values with different letter superscripts mean a significant difference (*p* < 0.05), and with different capital, letter superscripts mean an extremely significant difference (*p* < 0.01).

**Table 5:** Effects of concentrate supplementation on rumen immune factor content of yak calves in the suckling stage.

Term	RA	RAS	*p*-Value
IL-1β, ng/L	27.42 ± 1.40	28.94 ± 2.20	0.244
IL-2, ng/L	206.28 ± 10.23 ^b^	229.82 ± 13.69 ^a^	0.033
IL-4, ng/L	24.65 ± 2.64	27.13 ± 2.01	0.152
IL-10, ng/L	17.43 ± 1.77	17.92 ± 1.28	0.656
TNF-α, ng/L	160.94 ± 17.37 ^B^	254.69 ± 23.52 ^A^	0.005
TNF-γ, ng/L	114.89 ± 10.52 ^B^	170.58 ± 12.72 ^A^	0.001

In the same row, values with different letter superscripts mean a significant difference (*p* < 0.05), and with different capital, letter superscripts mean an extremely significant difference (*p* < 0.01).

**Table 6:** Differentially expressed genes of rumen.

Gene ID	Gene Name	Log2 FoldChange	*p*-Value
102278697	*LOC102278697*	1.17	0.002
102283235	*LOC102283235*	2.14	0.006
102274697	*PADI1*	1.37	0.002
102280156	*UPK1B*	2.45	0.001
102273667	*CYR61*	2.02	<0.001
102282243	*LOC102282243*	1.16	0.003
102266547	*ADAMTS1*	1.12	0.003
102270864	*LOC102270864*	1.18	0.04
102283377	*LOC102283377*	1.14	0.004
102271001	*SAMD4A*	1.16	0.002
102281124	*LOC102281124*	−1.96	<0.001
102271680	*S100A8*	−1.48	0.004
102274639	*S100A9*	−2.01	<0.001
102287056	*LOC102287056*	−2.01	0.004
102273665	*LOC102273665*	−2.40	0.01
102281207	*LOC102281207*	−3.25	0.004
106700804	*LOC106700804*	−2.43	0.02
102272590	*CENPF*	−1.03	0.001
102285929	*LOC102285929*	−2.23	0.03
102267775	*CFB*	−1.78	0.002

**Table 7:** Top 20 differentially expressed genes of jejunum.

Gene ID	Gene Name	log2 FoldChange	*p*-Value
ENSBMUG00000000550	*H1-4*	−1.42	<0.001
ENSBMUG00000011318	*CIDEC*	3.94	<0.001
ENSBMUG00000017645	*GPX2*	−2.58	<0.001
ENSBMUG00000026399	*PCBP3*	1.12	<0.001
ENSBMUG00000001962	*SNORD14*	1.27	0.001
ENSBMUG00000007647	*ACSS3*	0.85	0.002
ENSBMUG00000017694	*MYH13*	−4.33	0.002
ENSBMUG00000026770	*SNCG*	1.67	0.002
ENSBMUG00000008637	*ST8SIA6*	−3.57	0.002
ENSBMUG00000025733	*CACNG4*	−2.11	0.002
ENSBMUG00000026343	*ATXN7L3B*	0.49	0.003
ENSBMUG00000009103	*PIMREG*	−1.13	0.004
ENSBMUG00000000804	*HOXB9*	−4.10	0.004
ENSBMUG00000001028	*DLGAP1*	−1.25	0.004
ENSBMUG00000009241	*SFRP2*	−2.03	0.004
ENSBMUG00000013051	*CCNB1IP1*	−0.88	0.005
ENSBMUG00000012613	*TOGARAM2*	1.64	0.005
ENSBMUG00000024435	*H2BC11*	−1.28	0.006
ENSBMUG00000020980	*CMSS1*	−0.61	0.006
ENSBMUG00000007952	*SFTA2*	2.31	0.006

**Table 8:** Top 20 differentially expressed genes of colon.

Gene ID	Gene Name	log2FoldChange	*p*-Value
ENSBMUG00000018298	*CCL21*	−1.05	<0.001
ENSBMUG00000025335	*ITGAD*	0.91	<0.001
ENSBMUG00000026062	*GPR55*	0.98	<0.001
ENSBMUG00000023660	*FAIM2*	0.83	<0.001
ENSBMUG00000007952	*SFTA2*	5.47	<0.001
ENSBMUG00000015762	*CD247*	0.55	<0.001
ENSBMUG00000020941	*P2RY11*	0.77	<0.001
ENSBMUG00000015195	*CD6*	0.87	<0.001
ENSBMUG00000012590	*CCL5*	0.64	<0.001
ENSBMUG00000020282	*TRAT1*	0.53	<0.001
ENSBMUG00000025481	*MTFP1*	0.59	<0.001
ENSBMUG00000004572	*ACADSB*	0.78	<0.001
ENSBMUG00000025869	*NCOA2*	0.35	<0.001
ENSBMUG00000025786	*IGFBP2*	0.93	<0.001
ENSBMUG00000024641	*GZMB*	0.96	<0.001
ENSBMUG00000013012	*GALNT16*	0.79	<0.001
ENSBMUG00000004999	*GNL3*	−0.44	<0.001
ENSBMUG00000010409	*LRRC61*	0.67	<0.001
ENSBMUG00000020654	*HNRNPH1*	−0.24	<0.001
ENSBMUG00000025548	*SLC16A6*	0.67	<0.001

## Data Availability

The data supporting the findings of this study are available from the corresponding author upon reasonable request.

## Abbreviations

The following abbreviations are used in this manuscript:

ADF	acid detergent fiber
BP	biological process
Ca	calcium
CC	cellular component
CP	crude protein
DM	dry matter
DMI	dry matter intake
EE	ether extract
FBW	final body weight
FPKM	fragments per kilobase of transcript per million mapped reads
GO	gene ontology
IL-10	interleukin 10
IL-1β	interleukin 1 beta
IL-2	interleukin 2
IL-4	interleukin 4
KEGG	kyoto encyclopedia of genes and genomes
MCP	microbial crude protein
MF	molecular function
NDF	neutral detergent fiber
NH_3_-N	ammonia nitrogen
P	phosphorus
PCA	principal component analysis
SIgA	secretory immunoglobulin A
TFN-γ	tumor necrosis factor beta
TNF-α	tumor necrosis factor alpha
TVFA	total volatile fatty acids
VFA	volatile fatty acids
